# Eco-hydrology as a driver for tidal restoration: Observations from a Ramsar wetland in eastern Australia

**DOI:** 10.1371/journal.pone.0254701

**Published:** 2021-08-05

**Authors:** William Glamore, Duncan Rayner, Jamie Ruprecht, Mahmood Sadat-Noori, Danial Khojasteh

**Affiliations:** Water Research Laboratory, School of Civil and Environmental Engineering, UNSW Sydney, Sydney, NSW, Australia; Universidade de Aveiro, PORTUGAL

## Abstract

Land reclamation projects and the installation of drainage infrastructure has impacted coastal wetlands worldwide. By altering water levels and inundation extent, these activities have changed the viable ecosystems onsite and resulted in the proliferation of freshwater species. As more than 50% of tidal wetlands have been degraded globally over the last 100 years, the importance of this issue is increasingly being recognised and tidal wetland restoration projects are underway worldwide. However, there are currently limited sites where large-scale reintroduction of tidal flushing has been implemented with the explicit aim to foster the growth of a threatened ecosystem. In this study, the tidal restoration of an internationally recognised Ramsar listed wetland in eastern Australia is described to highlight how coastal saltmarsh can be targeted by mimicking inundation depths and hydroperiod across the 410-ha site. Coastal saltmarsh is particularly important to this site as it is part of the east Australasian flyway for migratory birds and the minimum saltmarsh extent, as listed within the Ramsar’s limits of acceptable change, have been breached. To recreate coastal saltmarsh habitat onsite, water level and hydroperiod criteria were established based on similar vegetation patterns within the adjacent estuary. A calibrated 2D hydrodynamic model of the site was then used to test how the preferred inundation criteria could be applied to the largest possible restored wetland area. Once optimised, a synthetic tidal signal was implemented onsite via automated hydraulic controls. The onsite vegetation response over an 8-year period was assessed to highlight the ecosystem response to controlled tidal inundation and denoted substantial saltmarsh expansion during the period. The techniques applied onsite have successfully met the restoration targets and can be applied at similar sites worldwide, offsetting sea level rise impacts to natural inundation hydroperiod.

## Introduction

Over the past century large-scale reclamation of tidal wetlands, including mud flats, mangrove, and saltmarsh ecological communities, have been undertaken worldwide [1, 2]. On a global scale, the loss of wetlands has been estimated at 50% since 1900 [3]. In some locations, reclaimed wetlands have provided productive agriculture, economic or greenhouse gas mitigation benefits [4–6], but in other areas this has resulted in a negative net outcome to ecosystem services or a direct environmental collapse [7–9]. The ecosystem loss from landscape reclamation has also significantly impacted habitat for aquatic and terrestrial fauna, including internationally protected migratory wader birds [10, 11].

In recognition of these issues, estuarine restoration projects have gained significant momentum worldwide [12–15]. In tidal environments this typically involves the reinstatement of tidal flows to re-establish intertidal ecological communities, rehabilitate sediments, and improve surface water and/or groundwater quality [16]. Restoration projects are also being undertaken to mitigate emissions and encourage carbon storage [17, 18] as methane production is limited when the salinity is above 17 ppt. Large-scale tidal restoration projects have been successfully undertaken globally, including the USA [19, 20], China [21–23], Korea [24], Europe [25], and Australia [26–28].

Over the past three decades, on-ground restoration methodologies and associated practices have significantly improved [29]. Initial methods were typically conducted using ‘trial-by-error’ techniques with limited ongoing monitoring or restoration goals [30]. This often resulted in poor community perception as ‘errors’ were perceived as poor techniques and/or the long-term goals were not analysed or achieved [31, 32]. However, several researchers have since encouraged restoration principles that ensure long-term monitoring of quantifiable end goals and performance criteria [32–35]. These principles are particularly important as the spatial extents increase, and more complex projects are attempted [15].

As rehabilitation or restoration projects become more commonplace, landscape managers are often beset with a sense of urgency to ‘do something’ onsite without detailed planning. Since hydrologic manipulations (e.g., floodgates, levees, culverts, etc) are often the cause of the initial site impact, the immediate response is to ‘restore’ onsite hydrology by removing these structures. However, what may initially appears as a linear ‘cause and effect’ mechanism (e.g., installed levee equals hydrologic isolation, therefore removal of levee equals good outcome) may lead to unexpected impacts, including second-order erosional effects, poor water quality runoff (e.g., blackwater), geomorphological changes, flooding impacts to adjoining landholders, and/or the enhancement of undesirable ecological communities [26, 36–38]. This may be compounded by broader estuary wide changes that have altered hydrologic (tidal prism, tidal planes) and/or ecologic (salinity regime) boundary conditions at the restoration site. In many locations these system-wide changes, and the associated ecological impacts, means that rehabilitation of the original ecological functional character is no longer feasible, and an alternative ecological regime would result if a ‘natural’ or the original hydrology is re-established.

Predictive tools or models used for analysing the rehabilitation or creation of tidal wetlands have evolved from simple conceptual models to GIS-based spatial models to complex eco-biological hydrologic-hydrodynamic response models [32, 39, 40]. While these models are well established to simulate hydrologic processes, based on known physical forcing mechanism, there is often limited data available to support the fundamental interrelationships between hydrology, ecology, and biology [40]. Therefore, in many circumstances the uncertainty of the predictive tools increases as they go from hydrologic simulations to ecologic predictions [41]. This is particularly important as most restoration performance criteria are based on ecologic criteria (e.g., desired species returning to site or the establishment of specific ecologic niche). This is further confounded by temporal, spatial, and ecosystem feedback loops [40].

This paper uses predictive hydrodynamic modelling to tackle challenges associated with rehabilitating or creating large tidal wetland environments where long-term ongoing management uncertainties must be minimised. To highlight this ‘front-loading’ approach, a 8-year case study of a large tidal wetland creation/restoration project in a Ramsar wetland site in south-eastern Australia is detailed. The ultimate objective of the restoration project was to increase migratory wader bird habitat onsite through the re-establishment of coastal saltmarsh communities, while minimising the colonisation of other undesired vegetation communities (e.g., mangroves). This was achieved by examining the existing inundation patterns within the adjacent estuary and attempting to replicate them onsite by controlling the tidal flushing dynamics. Other performance criteria included that (i) the proposed restored hydrologic regime is cost-effective, and (ii) does not negatively impact neighbouring landholders.

## Methodology

### Study site

**Fig 1:**
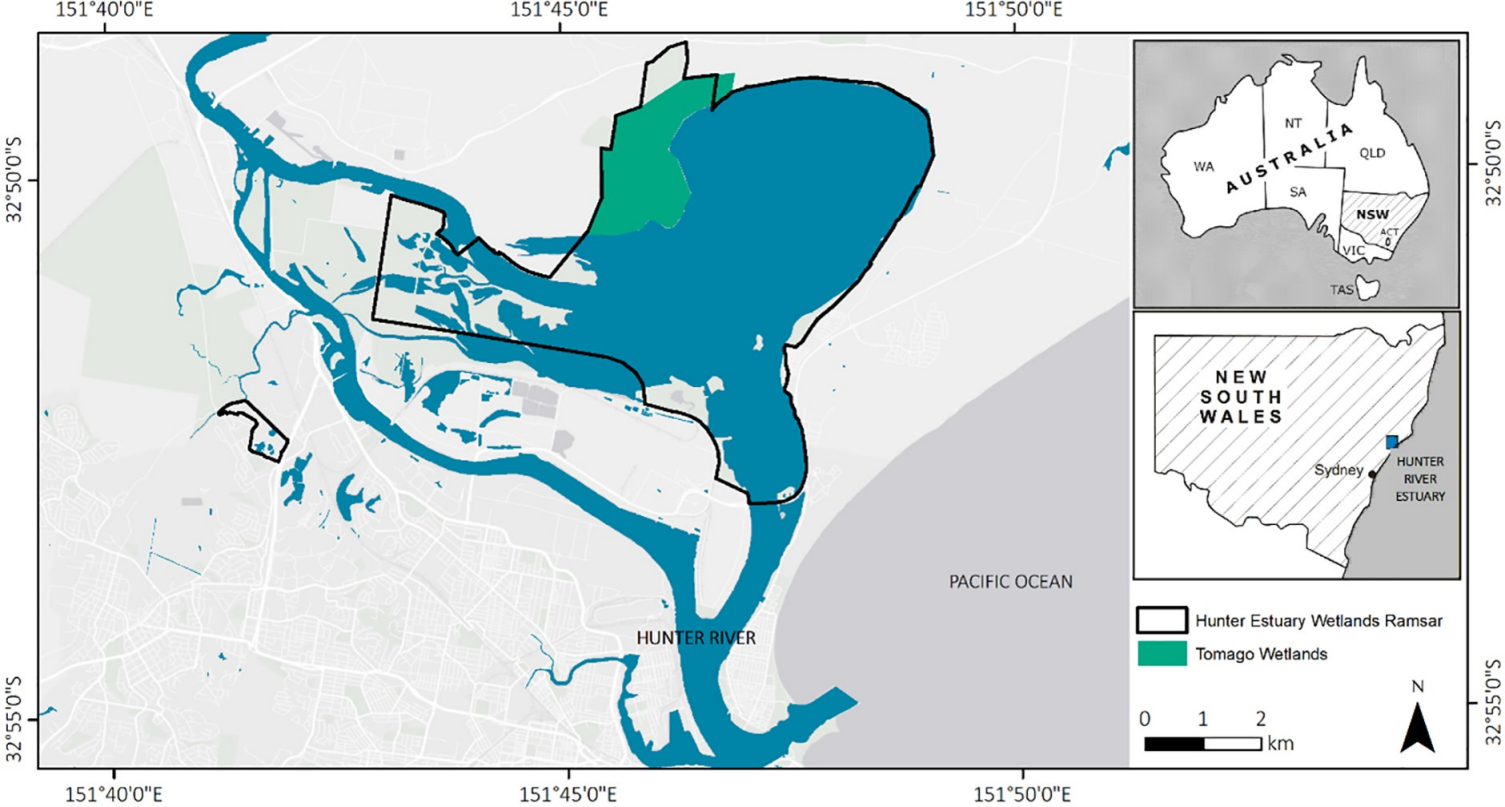
Location of the Tomago wetland restoration project and the Ramsar listed Hunter estuary wetlands.

Tomago Wetlands is located in the lower Hunter River estuary, a mature barrier estuary [42] on the central coast of New South Wales, Australia (Fig 1). The 410-ha site is located approximately 11 km from the micro-tidal ocean entrance at Newcastle. Tidal amplitude at the site varies from approximately ±1.2 m during spring tides to ±0.78 m during neap tides [43].

Between 1913 and 1928, a levee and internal drainage system was constructed around the perimeter of the site, which was subsequently enlarged by the NSW Public Works Department between 1968 and 1980 [44]. These engineering works, including the installation of floodgates at the tidal boundary, ensured that tidal waters were excluded from Tomago Wetlands (i.e., the site drains only via one-way tidal floodgates). The main objective of the levees and culverts was to provide flood mitigation for the lower Hunter River estuary (the ring levee overtops during a 2% Annual Exceedance Probability (AEP) flood event). During non-flood periods, dry-land agriculture was promoted but has subsequently failed [26, 45].

Drainage and the exclusion of tidal waters within the study site degraded the existing coastal saltmarsh habitat and fostered the growth of non-saltmarsh species [27]. Winning [46] demonstrated a change in species dominance onsite from coastal saltmarsh to the freshwater pasture species, *Paspalum dilatatum*. Lowering of the groundwater table also oxidised sub-surface acid sulfate soils causing soil acidification and poor water quality runoff (pH<4).

The Hunter Wetlands National Park (HWNP), including the Tomago Wetlands, was Ramsar listed in 1983. The site was listed based on (i) endangered and vulnerable species recorded onsite, (ii) its importance as a migratory shorebird foraging and roosting site, and (iii) regularly supporting more than 1% of the East Asian Australasian Flyway population of eastern curlew and Australian population of red-necked avocets. As part of the Ecological Character Description (ECD) for the Ramsar listing [47], Limits of Acceptable Change (LAC) were set for endangered and vulnerable species habitats onsite based on migratory bird counts, green and golden bell frog breeding events and the areal extent of coastal saltmarsh (minimum>466 ha). A review of the ECDs indicated that there had been a 41% decrease in the area of coastal saltmarsh to 339 ha across the HWNP, exceeding the LAC [47]. To compensate for the breach in the LAC, a major saltmarsh restoration project was commenced at Tomago Wetlands in 2007.

### Wetland restoration principles and objectives

The primary goal of the Tomago Wetlands restoration project was to recreate coastal saltmarsh above the LAC within the HWNP. This required the generation of 127 ha of suitable roosting and foraging habitat for migratory wading birds. This target aimed to limit the establishment of mangrove species within the restoration area, as migratory wading birds select for open water areas. Mangrove encroachment into coastal saltmarsh was recognised as a significant management concern in the lower estuary [48]. To this aim, the restoration principles outlined by Thom [35] and Simenstad, Reed [49] were adopted in relation to innovative adaptive management and integrated landscape connectivity. These outcomes were achieved through the development of a conceptual model, a detailed onsite monitoring and evaluation framework, an adjustment strategy, and the dissemination of information to local stakeholders.

### Regional wetland species distribution

**Fig 2:**
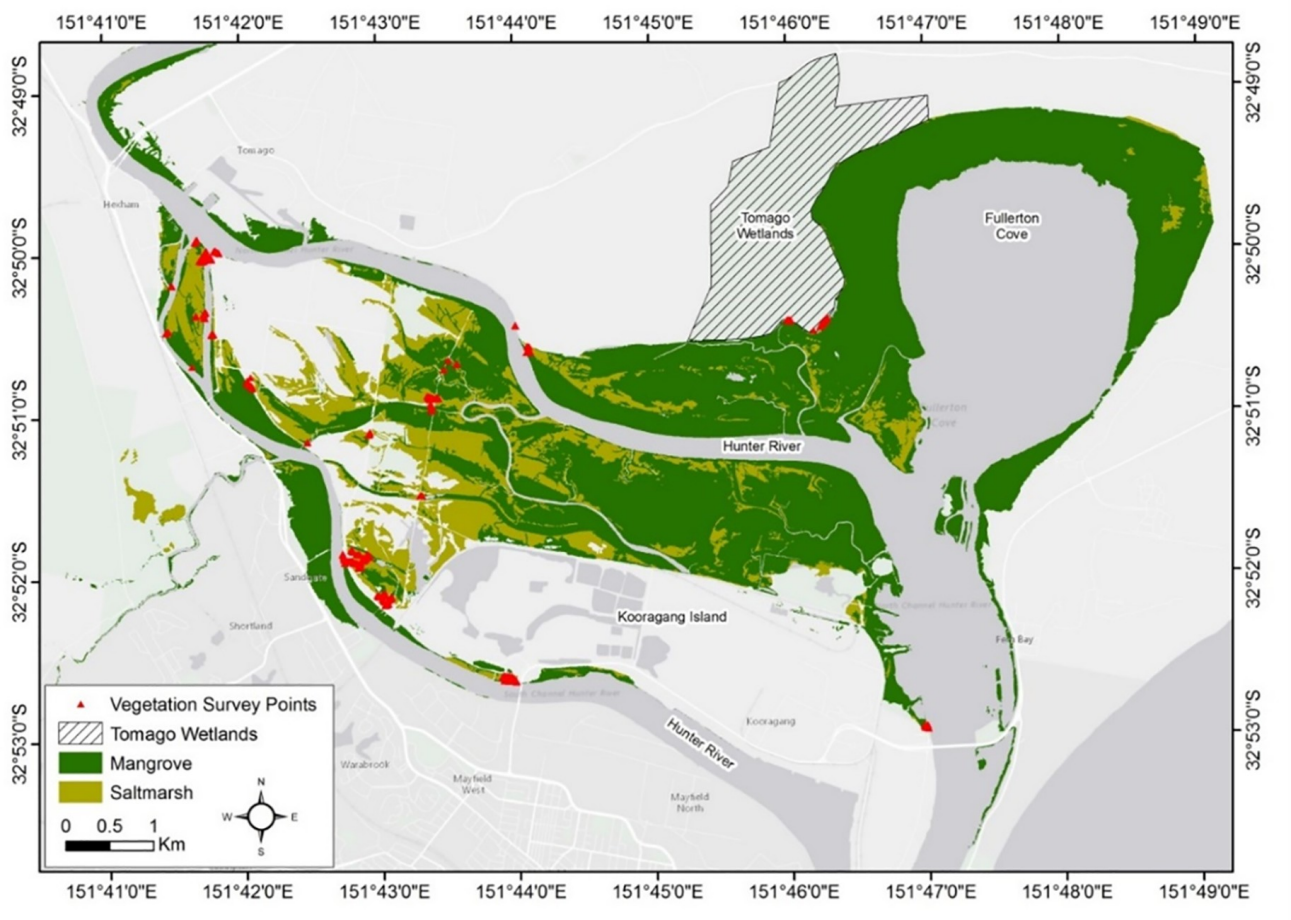
Coastal saltmarsh and mangrove survey locations (noted in red) in the lower Hunter River estuary (outside the restoration area). Regional mangrove and coastal saltmarsh mapping by Kleinfelder [53].

Restoration measures were guided by the associated risks of restoring tidal flushing onsite and the performance targets to increase coastal saltmarsh habitat. From an ecological perspective, tidal flushing could increase the colonisation of non-saltmarsh species, including mangroves and/or woodlands (e.g., Casuarina or Paperbark species). Howe [50] and Spencer and Howe [51] recommend limiting floodplain inundation to a depth below 0.3 m to promote the growth of coastal saltmarsh. Depths above 0.3 m were observed to significantly increase the establishment of mangrove habitat [51]. To confirm these inundation patterns, coastal saltmarsh and mangrove communities in the wider Hunter River estuary were surveyed in 2016 using a RTK-GPS (real-time kinematic global positioning system) to determine the ‘natural’ inundation regime of the functioning habitats (Fig 2). The vegetation elevations were then compared with nearby tidal plane measurements [43] to calculate the hydroperiod required to encourage saltmarsh habitat and limit mangrove encroachment onsite.

**Fig 3:**
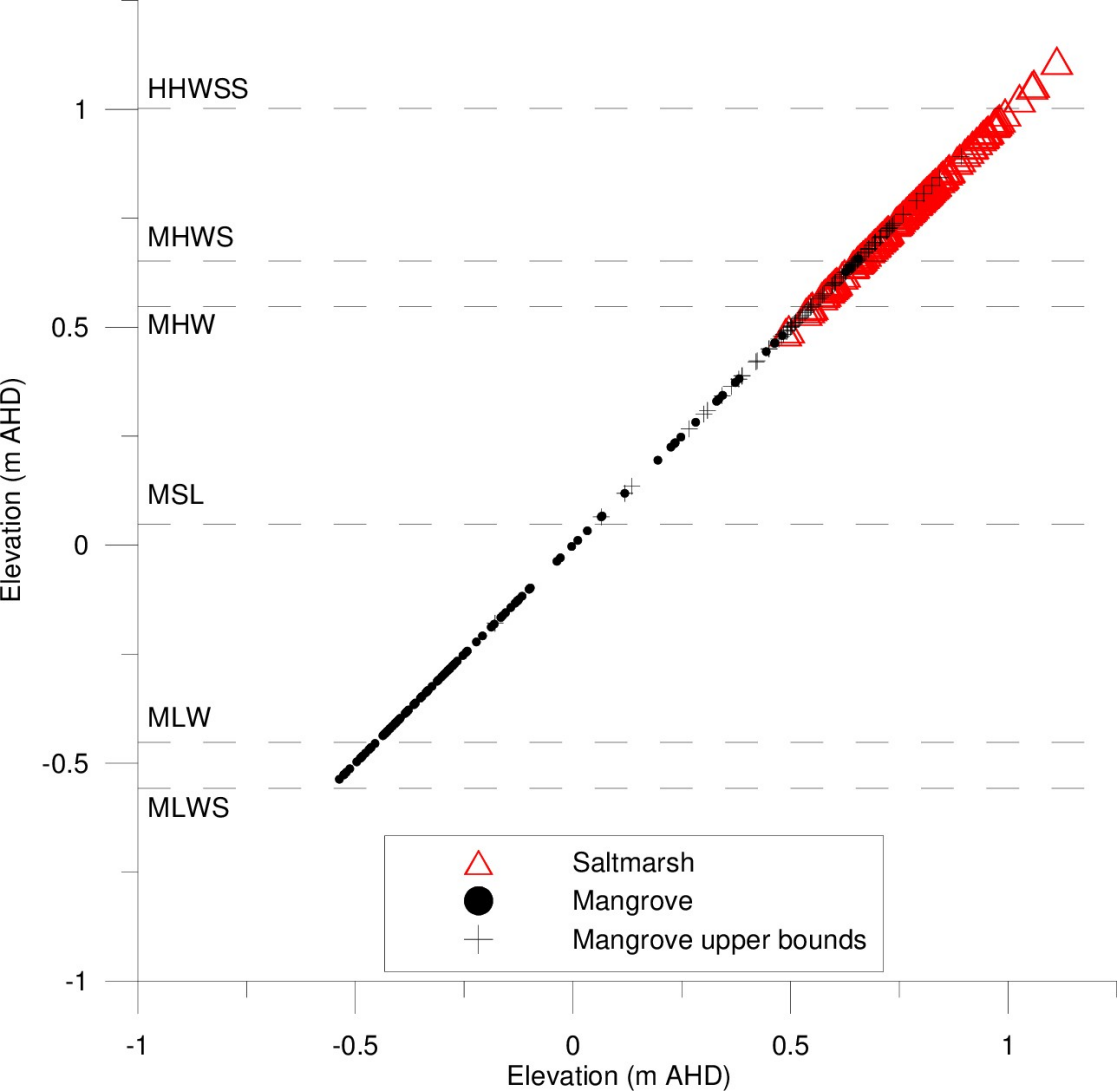
Surveyed elevations of coastal saltmarsh and mangrove elevations in the lower Hunter River estuary in relation to local tidal planes (MLWS = mean low water springs, MLW = mean low water, MSL = local mean sea level, MHW = mean high water, MHWS = mean high water springs and HHWSS = high high water spring solstice). All elevations relative to Australian Height Datum (AHD), where 0 m AHD is mean sea level.

**Fig 4:**
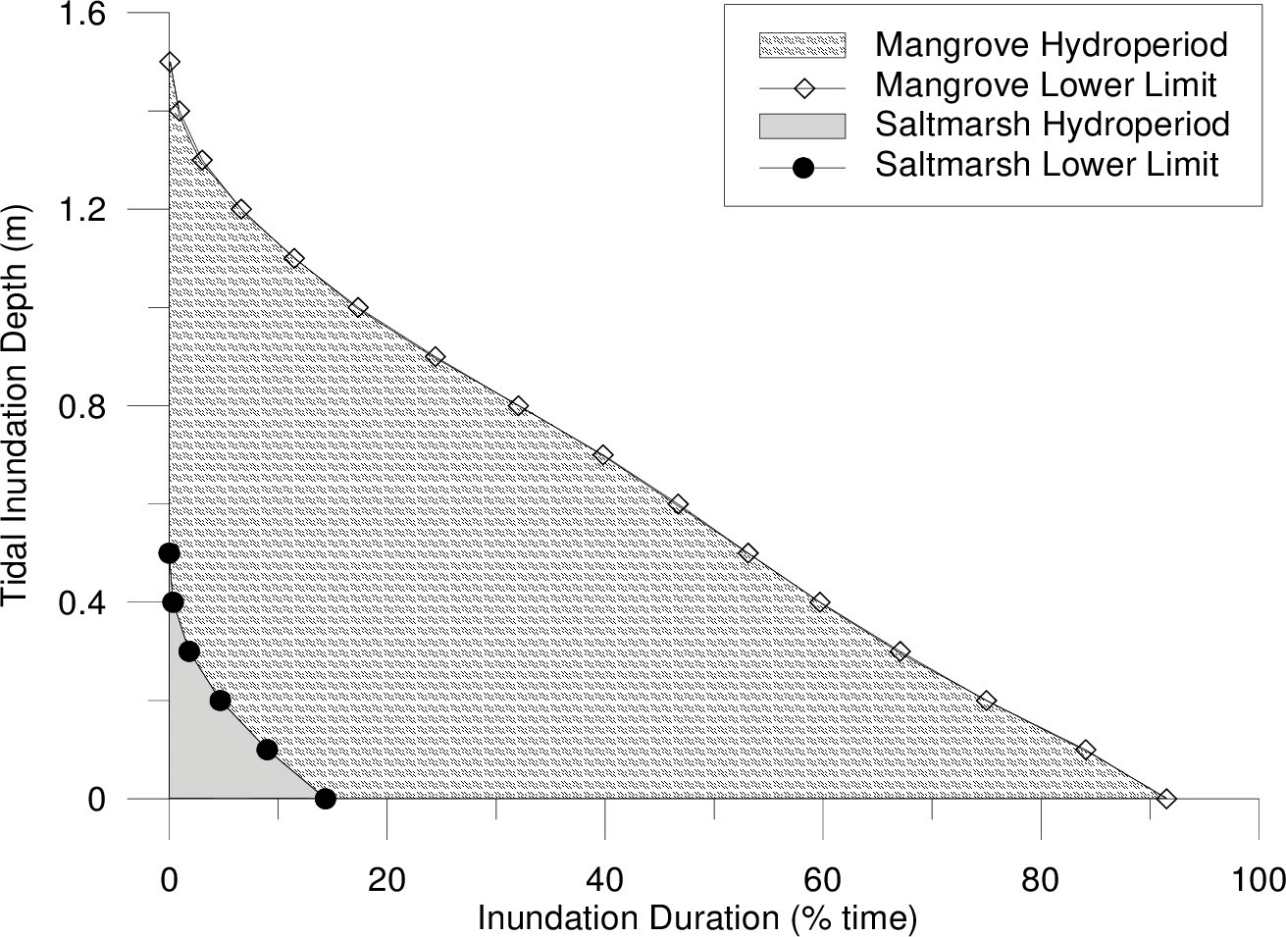
Hydroperiod (inundation duration) for surveyed mangrove and coastal saltmarsh communities in the lower Hunter River estuary based on local tidal planes.

Regionally, coastal saltmarsh dominates higher elevations in the tidal planes than mangroves. The majority of the coastal saltmarsh surveyed was found at elevations between local mean high water (MHW) (+0.55 m Australian Height Datum (AHD)) and high-high water spring solstice (HHWSS) (+1.00 m AHD) (Fig 3). While mangroves were predominantly observed at elevations below MHW. This results in depths of inundation below 0.4 m at all surveyed coastal saltmarsh communities (Fig 4). Species encroachment was observed at elevations above and below these tidal planes.

Fig 4 summarises the tidal hydroperiod (inundation depth and duration) for reference vegetation sites and/or modelled scenarios. When determining the hydroperiod of mangroves and saltmarsh vegetation at reference sites, the measured tidal signal (at a 15-minute interval) compared with the surveyed ground surface elevations at existing mangrove and saltmarsh vegetation communities were analysed to determine the tidal hydroperiod statistics for each intertidal vegetation community. This analysis is repeated across surveyed reference sites to determine the range of hydroperiods relating to different naturally occurring saltmarsh and mangroves. When analysed over all possible tidal ranges (for example over a full spring-neap tidal cycle (or longer)), inundation depth:duration statistics can be developed for each topographic point (which corresponds to different observed vegetation communities). For reference vegetation communities, this analysis was completed at 0.1 m depth intervals to identify the inundation depth and duration statistics where mangroves or saltmarsh are likely to grow. The hydroperiod statistics for all surveyed mangroves and all surveyed saltmarsh communities are collated to establish a range of potential inhabitable hydroperiods.

### Hydrodynamic modelling

A hydrodynamic model developed by Rayner and Glamore [26], and further detailed in Rayner, Glamore [27], was applied to simulate scenarios of existing conditions and restored tidal flushing to estimate the risks/benefits associated with each scenario. The reader is directed to [26] as well as the Supporting Information for a description of the model set-up and calibration procedures. A brief description of the model parameterisation is provided below for reference for this study.

The commercially available MIKE FLOOD [52] model was employed to simulate 1D/2D channel and overbank flows as well as wetting/drying cycles. MIKE 11 was used to simulate 1D flows in the main channel network, including culverts and floodgates, whereas MIKE 21 was used to simulate 2D overland flow and marsh areas. MIKE FLOOD linkage elements coupled the two models to provide a hydrodynamically linked model.

Field data was collected including water levels, channel discharge, topographic and bathymetric data. This data was used to calibrate the hydraulic flows through culverts, ground-truth LiDAR data across the study domain, determine bathymetric profiles of the existing channel network, and assess onsite hydraulic roughness. The data was also used to develop a conceptual model of tidal restoration onsite, which was reviewed by local stakeholders.

The drainage network within the model was simulated as a series of linked branches with the flow dependent on drain geometry and structures. Cross-sectional surveys were undertaken using signal corrected Trimble RTK-GPS survey equipment accurate to ±20 mm and a single frequency echo sounder. Six major hydraulic structures were simulated using invert and structural dimensions as surveyed for this study. The site is not subject to upland catchment inflows.

The 2D model topography was sourced from airborne LiDAR data flown during a dry period (i.e., prior to tidal restoration). Additional ground-truthing was undertaken to ensure there was no bias in the LiDAR data and that accurate topographic information was available at the 1D/2D intersections. The average elevation of the restoration site was calculated as 0.1 m above MSL. The applied grid resolution resulted in approximately 380,000 grid points across the 410-ha site.

Model roughness was based on local measurements of Howe [50] for saltmarsh and pasture grass, in combination with topographic and the vegetation maps. Areas with shallow surface water inundation were assigned a Manning’s *n* of 0.1, with drainage channels set at 0.03 and dense stands of *Phragmities australias* set at 0.25.

A time series of recorded water levels were applied as the tidal boundary conditions for both model calibration and scenario testing. The water level measurements were sampled at 15-minute intervals using Solinst water level loggers, adjusted for barometric pressure. A Sontek Argonaut-SW was installed to measure bi-directional discharge over the same period every 15 minutes. Field experiments were undertaken where the tidal front was mapped hourly across the landscape using handheld RTK-GPS markers each hour during a flood tide to verify the movement of shallow tidal water across the wetland. This information was used to refine the stage-volume relationship for the site as developed from the ground-truthed LiDAR. All measurements were corrected to Australian Height Datum (AHD).

Simulations were run at a timestep (Δt) of 2 seconds to maintain a Courant number < 1. Model simulations were completed over a (minimum) 28-day spring-neap tidal cycle to ensure all potential tidal inundation depths and durations (i.e., hydroperiod) were predicted. A ‘hot start’ initial condition was used to ensure the model had sufficiently reached a mass balance equilibrium. Model results were output at an interval equal to, or greater than, the model timestep. Model results over the 2D domain were exported every 5 minutes (150 timesteps) and statistical analysis of predicted inundation (extent and hydroperiod) was completed and compared to reference site vegetation hydroperiod data.

Each stage of the restoration was modelled iteratively to adjust management of the key hydraulic control structures (i.e., reduce or increase tidal signal) to achieve optimised restoration outcomes, targeting saltmarsh vegetation establishment and minimal impacts to adjacent landholders. Further details regarding the numerical method, boundary conditions, and validation are provided in [26, 27] as well as in S1–S3 Figs.

### Restoration scenarios

**Fig 5:**
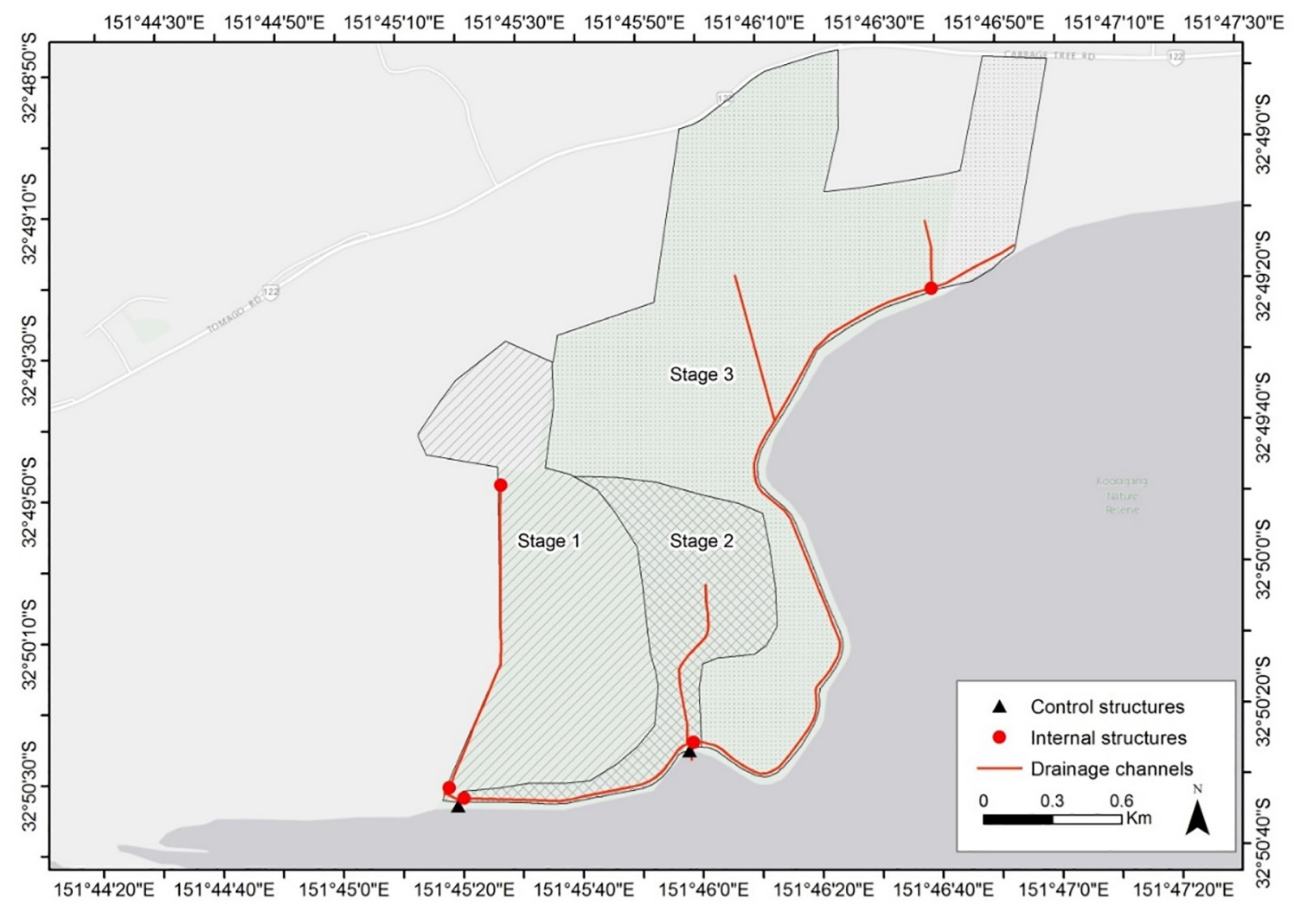
The primary drain network featuring internal hydraulic structures that allow for a staged restoration approach.

A range of engineering modifications were simulated to ‘restore’ tidal flushing onsite. In its pre-restoration state, tidal flushing was restricted due to two series of culverts located in the southwest (5 x 1.8 m diameter culverts) and southeast (4 x 1.8m diameter culverts) of the site. In addition, floodgates and open culverts redirect flow between internal drainage lines (Fig 5). Physical modifications to the floodgates on these culverts to permit flow exchange was a critical part of the overall restoration plan.

The other primary risk to the project was any potential inundation of adjacent private land. This was particularly related to adjacent landholders not associated with the project who must maintain their existing drainage. For these locations, the modelling scenarios determined the extent and design of on-ground engineering works, including levees and culverts to offset the inundation risk. The scenario that resulted in the largest extent of predicted saltmarsh habitat, with the minimal amount of adjoining land risk, was determined to be the ideal scenario for implementation.

Restoration was undertaken using a staged approach. The three stages of restoration were implemented over an 8-year period, with Stage 1 opened in November 2007, Stage 2 in September 2011, and Stage 3 in December 2015 (Fig 5). The restoration strategy and scenario testing were designed such that all stages of restoration were considered to ensure optimal inundation hydroperiods across the site to target saltmarsh species.

**Fig 6:**
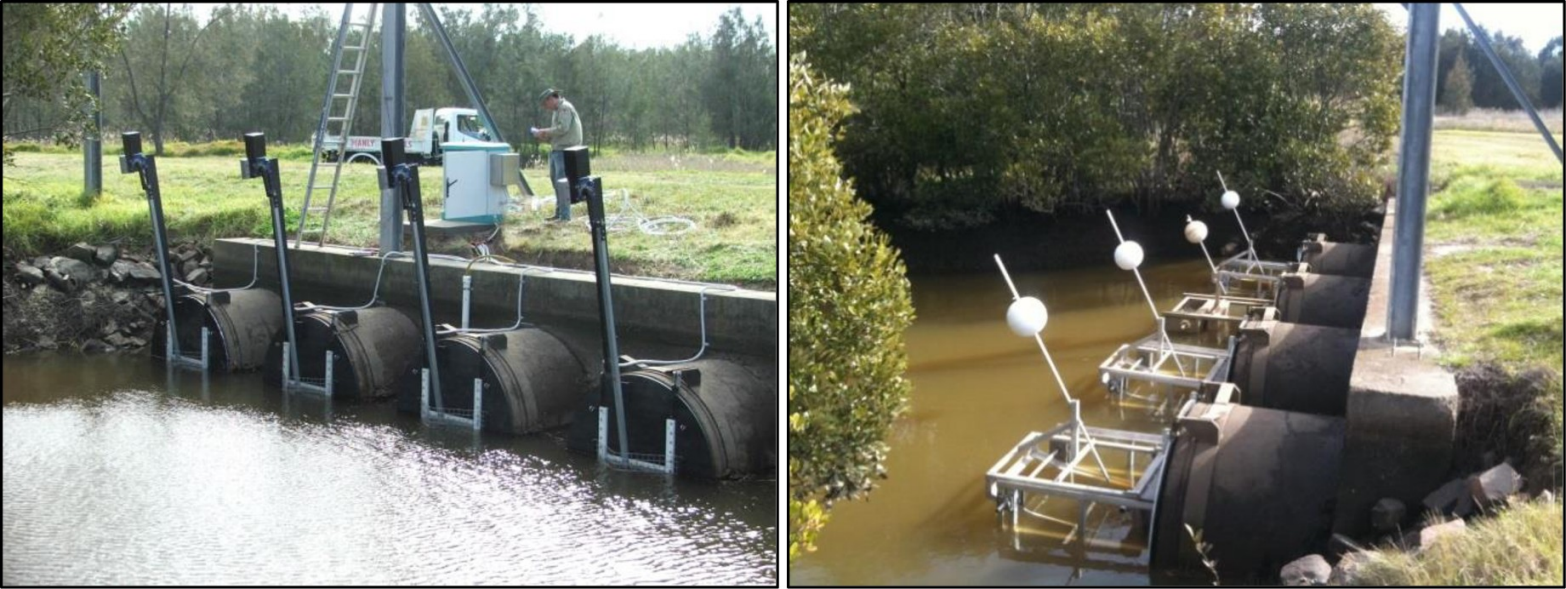
Electronic SmartGates at Stage 1 (left) and buoyancy controlled SwingGates at Stages 2 and 3 (right).

Stage 1 of the restoration involved the construction and installation of electronic SmartGates [37] to enable accurate wetland water level control (Fig 6), clearing of in-drain freshwater weed vegetation species to promote efficient tidal flushing, isolation of the Stage 1 area by installing one-way floodgates on internal drain culvert structures, and construction of low levees to limit the impact of tidal flushing on adjacent private land holders. Removal of exotic vegetation and mangroves was also undertaken immediately prior to tidal restoration. Water levels and salinities in adjacent land and connected waterways were monitored to ensure impacts to adjacent landholders were minimised.

Following the restoration of tidal flushing, water levels, water quality, fisheries, and wetland vegetation response were routinely monitored across the Stage 1 restoration area. Based on the monitoring, tidal flushing of Stage 2 was restored via the installation of buoyancy controlled SwingGates (Fig 6). The SwingGates were designed to minimise stress to aquatic fauna by maintaining ambient flow velocities through the gate aperture. Unlike traditional buoyancy controlled tidal floodgates, the SwingGates operate in either a fully open or fully closed condition, thereby reducing drag on the gate, as well as maximising the opening area during operation. Stages 2 and 3 are separated by natural topographic features and internal floodgated culverts. Stage 3 was commissioned by the modification of standard floodgates to incorporate a manually operated orifice sluice gate. This structure is located immediately upstream of the Stage 2 buoyancy controlled SwingGates and regulates water levels within the Stage 3 restoration area.

Hydroperiod predicted by the model results was compared to observed vegetation at the reference sites (Fig 4). This was used to assess the vegetation likely to establish under the modelled hydrological conditions over a full spring-neap tidal cycle (or longer) at the Tomago Wetlands.

## Results and discussion

### Tidal water level

**Fig 7:**
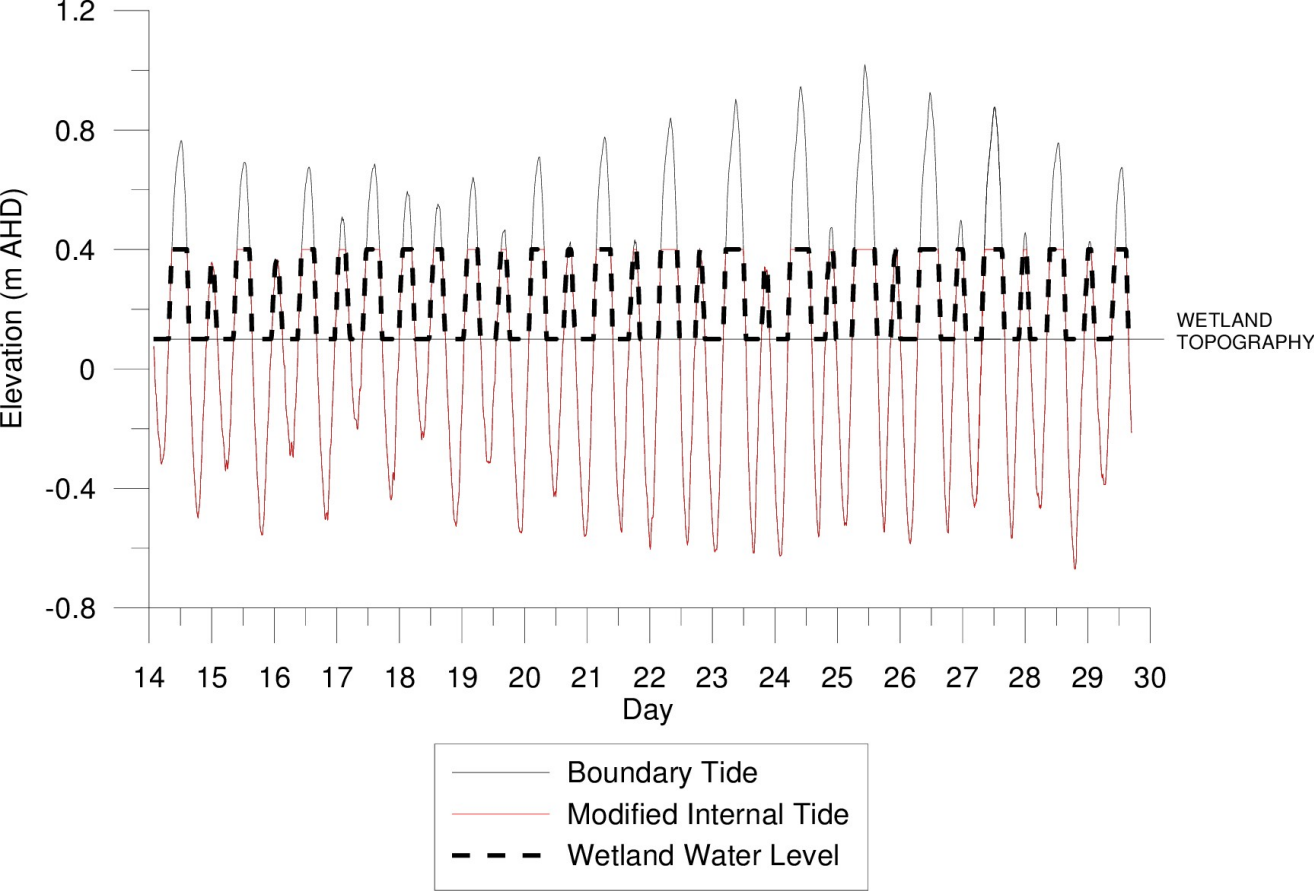
Example time series of observed downstream tidal boundary, the required muted tidal signal based on wetland topography and saltmarsh hydroperiod, and the predicted water level within the wetland.

Based on inundation statistics of wetland vegetation surveyed across the lower Hunter River estuary a maximum inundation depth of 0.3 m was targeted in the restored wetland to promote coastal saltmarsh. Initial modelling simulations indicated that unrestricted tidal flushing across the site would impact neighbouring properties and result in hydrologic conditions that are favourable to mangroves, not coastal saltmarsh. As this exceeded the pre-set design criteria, a series of simulations were undertaken where the tidal inundation was muted, via auto-tidal floodgates at the tidal boundary, to reduce the inundation depth and extent. Within the numerical model, the auto-tidal floodgates muted the boundary tidal signal to ensure a peak water level of +0.4 m AHD within the wetland. Fig 7 represents these boundary conditions applied in the model and onsite, in contrast to the naturally occurring tides (without any restrictions).

**Fig 8:**
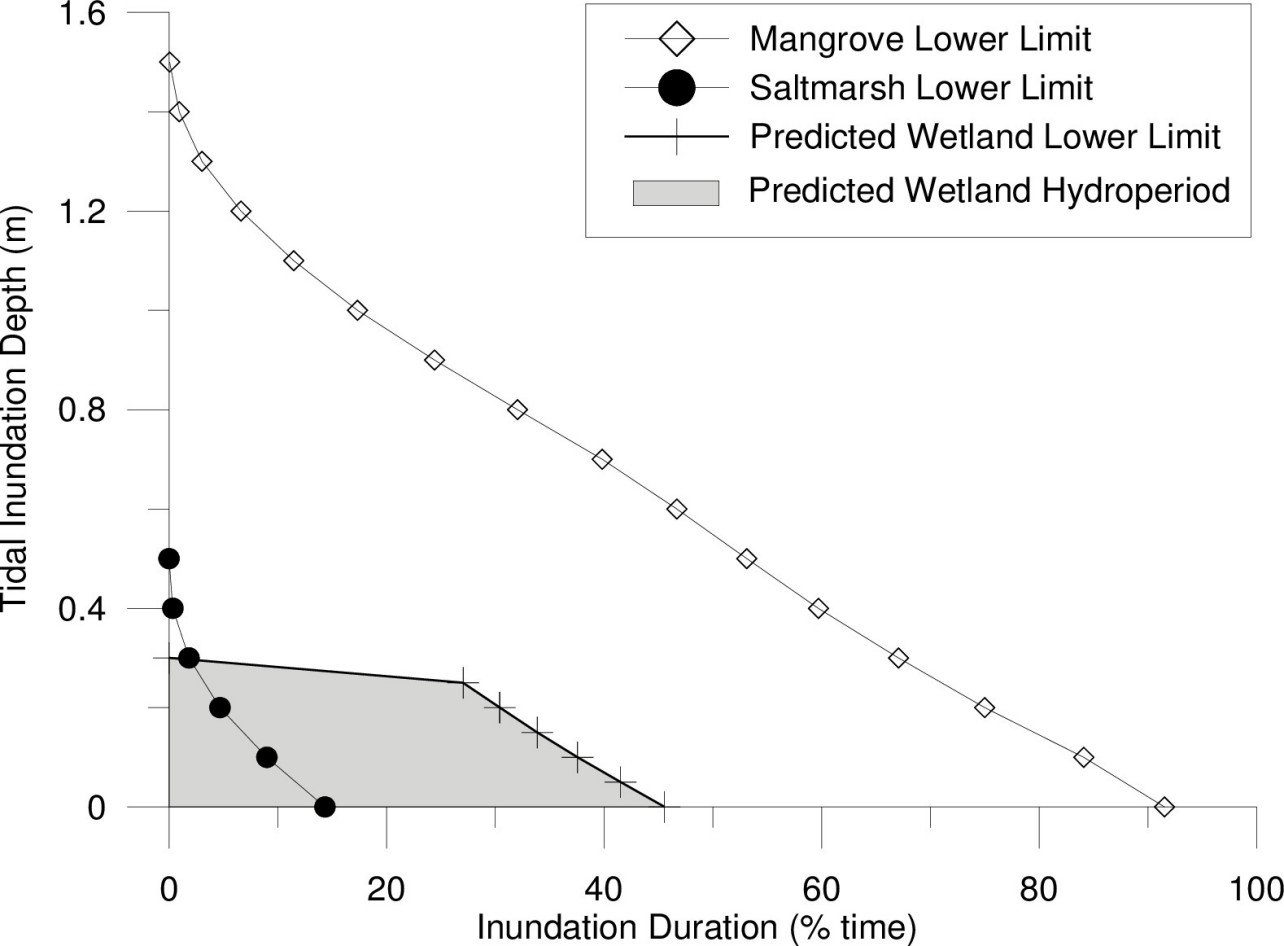
Predicted wetland hydroperiod based on the muted internal tidal signal versus desired hydroperiod for saltmarsh species.

Theoretical inundation statistics likely to occur across the restoration site were calculated based on the muted tidal signal (Fig 8). These statistics indicated that whilst inundation depths were not predicted to exceed 0.3 m, as observed in the estuary wide vegetation survey, the duration of inundation was expected to increase. This was due to the muted tidal signal that produced an extended period of inundation at the maximum acceptable cut off elevation of +0.4 m AHD, whilst the downstream natural tidal signal rises above the cut off elevation. Reducing the inundation duration would require the entire wetland topography to be at a higher elevation, or the tidal boundary elevation to be lowered, both of which were not feasible.

### Tidal inundation

For each restoration stage, water levels across the site were tested against the desired ecological targets and the required infrastructure necessary to achieve the targets. As a reminder, the stages were designed to be opened in succession, with the Stage 3 scenario incorporating active restoration across both Stage 1 and Stage 2 areas. This was particularly important as the inflow of tidal waters between stages resulted in hydraulic interactions onsite.

Modelling results indicated that the desired outcomes could be achieved onsite for a range of scenarios, but that onsite planning and engineering was required. A full list of inundation extents and volumes for each stage is provided in Table 1. Similar inundation areas and volumes for Stages 2 and 3 indicate that the proposed auto-tidal floodgates, which regulate tidal elevation and flushing onsite, were important for the hydraulic control for the eastern areas of the site (Stages 2 and 3).

**Table 1:** Tidal inundation model results.

Scenario	Inundation Area (ha)	Inundation Volume (m^3^)
Stage 1	33.7	9,000
Stage 1 + Stage 2	50.7	23,000
Stage 1 + Stage 2 + Stage 3	131.4	22,000

**Fig 9:**
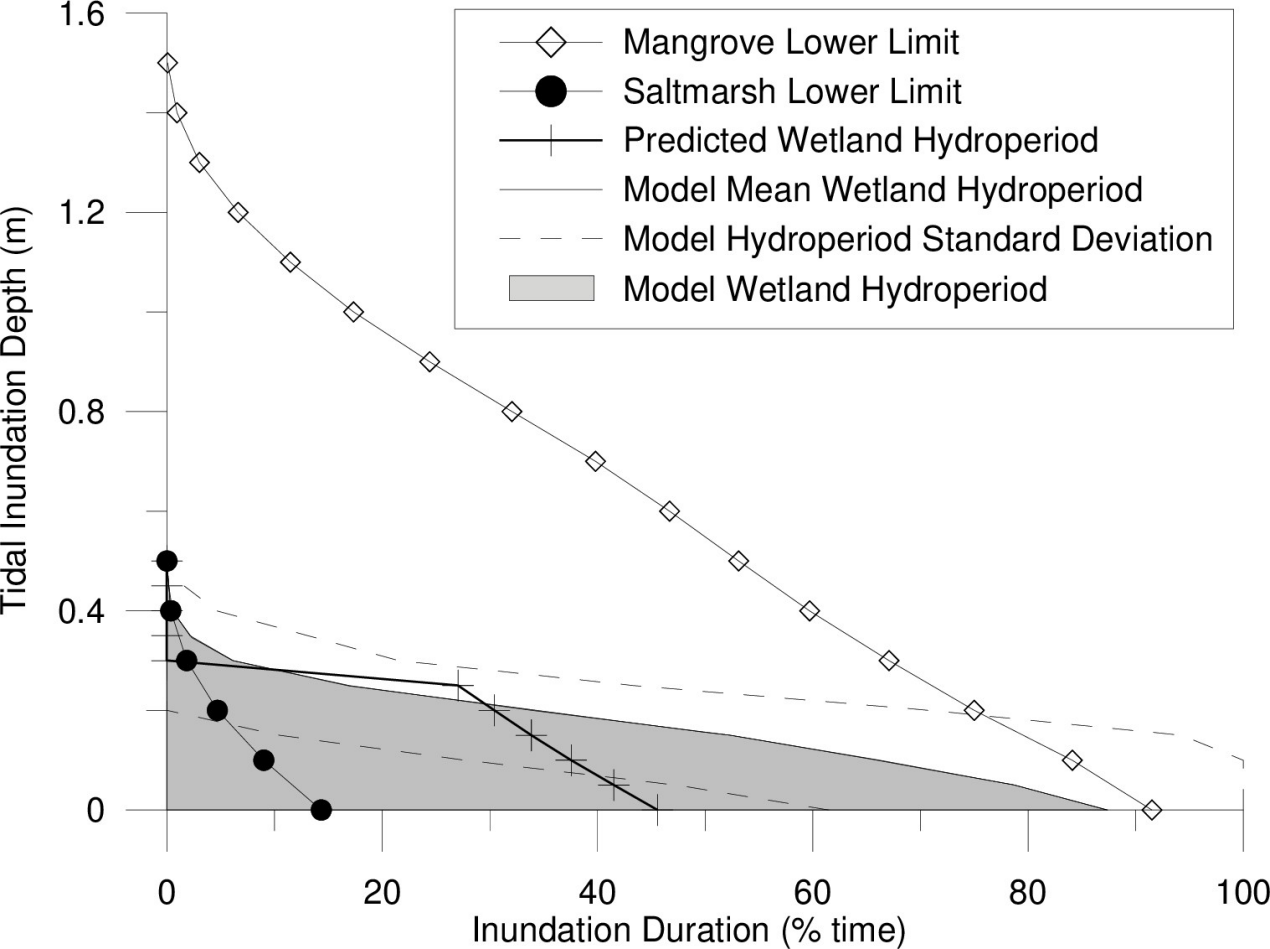
Predicted wetland hydroperiod based on a muted internal tidal signal.

2D inundation results were extracted from the numerical model and hydroperiod statistics calculated for wet areas only. When compared to the theoretical inundation depth based on the water level time series only, the model results indicated similar maximum inundation depths of 0.3 m depth (Fig 9). Maximum inundation depths were modelled up to depths of 0.4 m in some locations. This was due to the natural irregular topography across the site. However, the model predicted an overall increase in inundation duration, which was due to the flat site topography resulting in poor connectivity at shallow water depths. In turn, this produced disconnected shallow pools and open water in some locations. Inundation duration at shallow depths could be reduced if the site had a defined slope and elevation gradient (i.e., with laser levelling).

A range of hydroperiods were also simulated in the numerical model. Although the restoration site is predominately flat, varying topography across the site resulted in different inundation depths and durations. Additionally, tidal overland flow characteristics resulted in varying spatial inundation statistics, with areas directly adjacent to drains having a faster filling and draining time compared with areas at distance from the main channels. These features highlight the value of a hydrodynamic approach (versus a static GIS approach) and resulted in a range of modelled hydroperiods that may be applicable onsite.

### Vegetation changes

The distribution of vegetation communities was surveyed before, during and after the restoration project (Table 2). Mapping was undertaken by Winning [46], OEH [54], Kleinfelder [55], and Kleinfelder [53] using standard aerial imagery and ground-truthed methods including transect and point survey techniques. Additional methodological descriptions are provided in [27, 53–55]. Prior to restoration, the site was dominated by pasture and grass species, with no wader bird habitat observed. Following the commission of Stage 1 and Stage 2 tidal flushing, vegetation mapping in June 2012 showed species replacement with an 85% reduction in pasture and exotic species, and a significant increase in coastal saltmarsh and intertidal pools/ponds from 0 ha to 72 ha and 14 ha, respectively. Due to the June 2012 survey being completed soon after the commissioning of Stage 2 restoration area (September 2011), the vegetation distributions represented in this survey were predominately a result of the Stage 1 restoration phase.

**Table 2:** Vegetation extents and restoration timeline.

Vegetation Type	1993^#^	Dec-2008	Sept-2011	June-2012^ˆ^	May-2015*	Nov-2015	May-2016*
Mangroves (ha)	6	Stage 1 Restoration	Stage 2 Restoration	0	3	Stage 3 Restoration	2
Saltmarsh (ha)	0			72	85		85
Brackish Grasses/Sedgeland (ha)	55			72	21		21
Phragmites Australis/Reed (ha)	22			91	95		94
Swamp Oak, Casuarina, and Paperbark (ha)	15			83	81		80
Pasture and Exotics (ha)	256			40	62		55
Intertidal Mudflats and Shallow Ponds (ha)	0			14	62		62

^#^Estuary wide mapping by [46] adjusted to match methodology by [53, 55]

^^^ Mapping by [54]

* Mapping by [53, 55]

Aerial vegetation mapping in May 2015 reflected changes due to the Stage 1 and 2 restoration areas. In comparison to vegetation mapping following Stage 1 restoration, the May 2015 survey indicated an 18% increase in coastal saltmarsh from 72 ha to 85 ha, and a 440% increase in intertidal mudflats and shallow ponds from 14 ha to 62 ha. The change in species dominance was due to the evolution of the Stage 1, as well as Stage 2 transitioning to a tidal saltwater system.

Vegetation mapping undertaken within 6 months of Stage 3 indicated similar community composition patterns as observed in Stage 2 in May 2015. This was likely due to the response rate of the vegetation to tidal restoration, with existing freshwater/brackish species within the Stage 3 restoration area. These findings indicate that following tidal restoration, species replacement should occur after 6 months, with significant changes in species dominance and composition after 4 to 5 years.

These results indicate that tidal restoration using hydrologic controls can successfully recreate ecologically critical coastal saltmarsh habitat in a previously drained landscape. The findings have been further supported by large sightings of migratory shorebirds (not previously recorded), including in excess of 3000/month records of a target species, sharp-tailed sandpiper, onsite from 2014 onwards [56]. This is more than 1% of the global population of the shorebird species and an internationally significant outcome for the project [57]. Interestingly, significant sightings were not recorded until approximately 5 years after the tidal restoration project, which may imply a lag between coastal saltmarsh growth and benthic infauna response sufficient to attract shorebirds onsite.

While the methods and hydraulic controls implemented onsite suggests that hydrologic manipulation can effectively mimic natural inundation patterns (Fig 3), these patterns are threatened by rising sea levels. As per the modelling results, maximum water levels may be manipulated onsite to replicate maximum water levels in the estuary, but this is only effective when the natural landscape features that control inundation are not breached by the rising tide. Further, the hydroperiod analysis (Fig 4) highlights that the raising of the entire tidal range (not just the high tide) is more difficult to control and is likely to result in prolonged periods of inundation or reduced drainage. Further research is required to understand how the landscape would respond to these altered conditions.

## Conclusions

Despite years of global decline the importance of coastal wetlands has been increasingly recognised [58, 59]. In recent years, different techniques have been implemented to restore remnant coastal wetlands or recreate lost habitat [2, 60, 61]. To date, however, limited research and on-ground applications have been undertaken to bio-mimic the physical inundation patterns of desired existing habitat of nearby habitat to large areas of potential habitats using eco-engineering techniques.

As a drained floodplain that was once a large tidal wetland, Tomago Wetlands is an ideal test site to implement the saltmarsh bio-mimicry eco-engineering methods described in this paper. The test site is representative of many other locations worldwide and typical of the types of wetlands previously reclaimed over the past century. The study highlights that detailed eco-hydrologic design methods for tidal wetlands, supported by targeted field data campaigns and numerical models, can be used to mimic ecological responses and foster ecosystem restoration.

Restoring or recreating tidal ecosystems is gaining increasing importance worldwide and the United Nation has declared 2021–2030 the Decade of Ecosystem Restoration. As detailed in this study, the successful implementation of large-scale on-ground ecosystem restoration requires moving beyond a ‘trial by error’ approach and towards a systematic and tested method that integrates multi-disciplinary science and extended monitoring timeframes (e.g., beyond 5 years). As future coastal restoration or recreation projects are also likely to be managing sea level rise pressures, the bio-mimicry approach detailed wherein may also be a feasible method to temporarily abate coastal squeeze [15].

## Supporting information

S1 Fig Locations of restoration area in the Tomago wetlands and the water level and flow monitoring site. (PDF)

S2 Fig A comparison between the predicted and measured water levels at the flow monitoring site. (PDF)

S3 Fig A comparison between the predicted and measured flow rates at the flow monitoring site. (PDF)
