# Supplementary material for: Eco-hydrology as a driver for tidal restoration: Observations from a Ramsar wetland in eastern Australia
Source: PLoS One. 2021 Aug 5;16(8):e0254701. doi: 10.1371/journal.pone.0254701 (PMC8341630; doi:10.1371/journal.pone.0254701)

S1 Fig. Locations of restoration area in the Tomago Wetlands and the water level and flow monitoring site. Note that all tidal waters entering or leaving the restoration area must flow via the flow/level monitoring location.

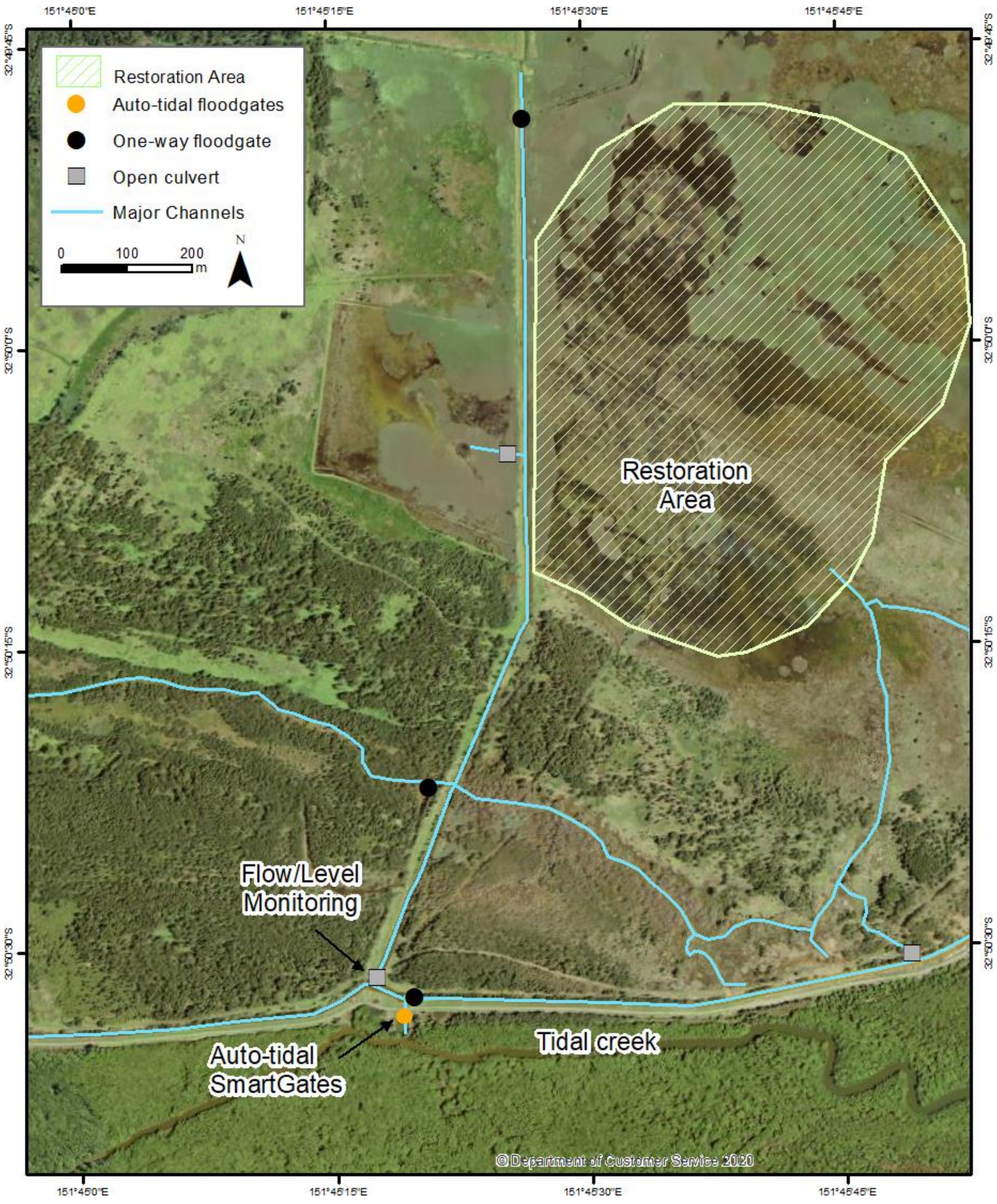

Supplement: S1 Fig — (PDF) [file pone.0254701.s001.pdf]
