# Supplementary figures and images for: Eco-hydrology as a driver for tidal restoration: Observations from a Ramsar wetland in eastern Australia
Source: PLoS One. 2021 Aug 5;16(8):e0254701. doi: 10.1371/journal.pone.0254701 (PMC8341630; doi:10.1371/journal.pone.0254701)

**S2 Fig.** A comparison between the predicted and measured water levels at the flow monitoring site.

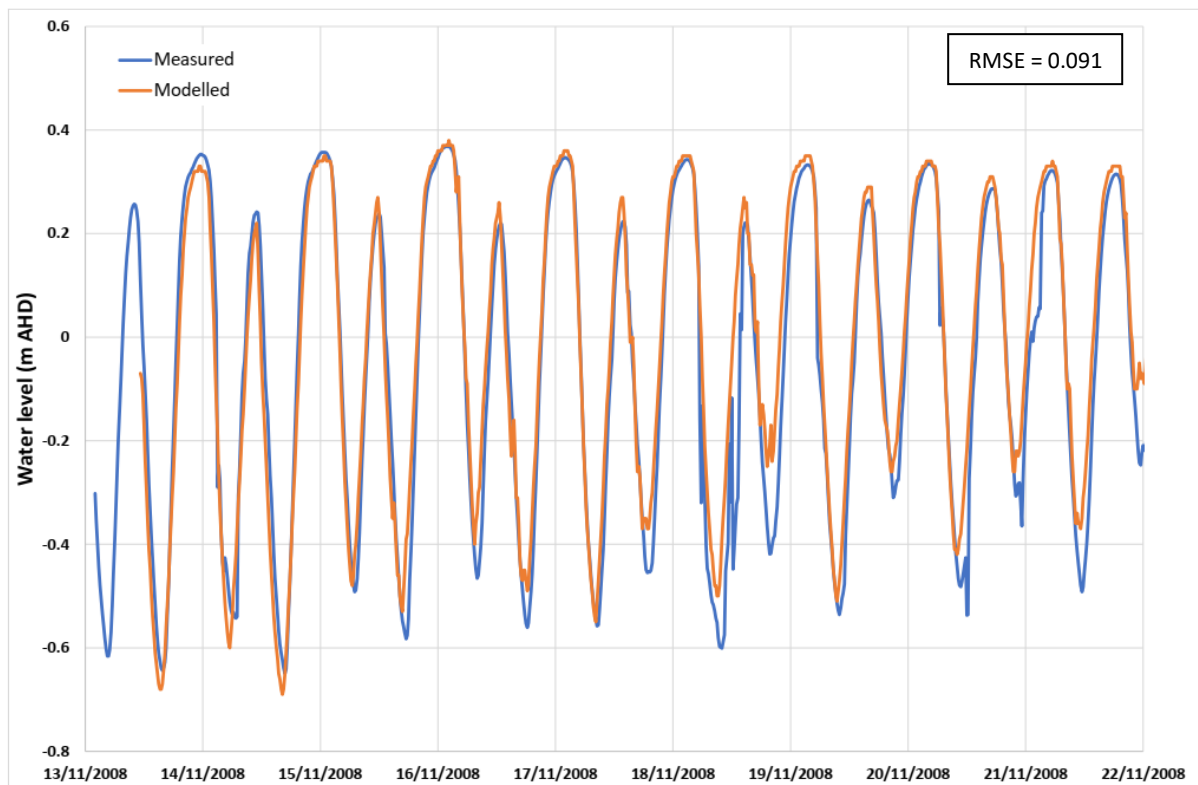

Supplement: S2 Fig — (PDF) [file pone.0254701.s002.pdf]

**S3 Fig.** A comparison between the predicted and measured flow rates at the flow monitoring site.

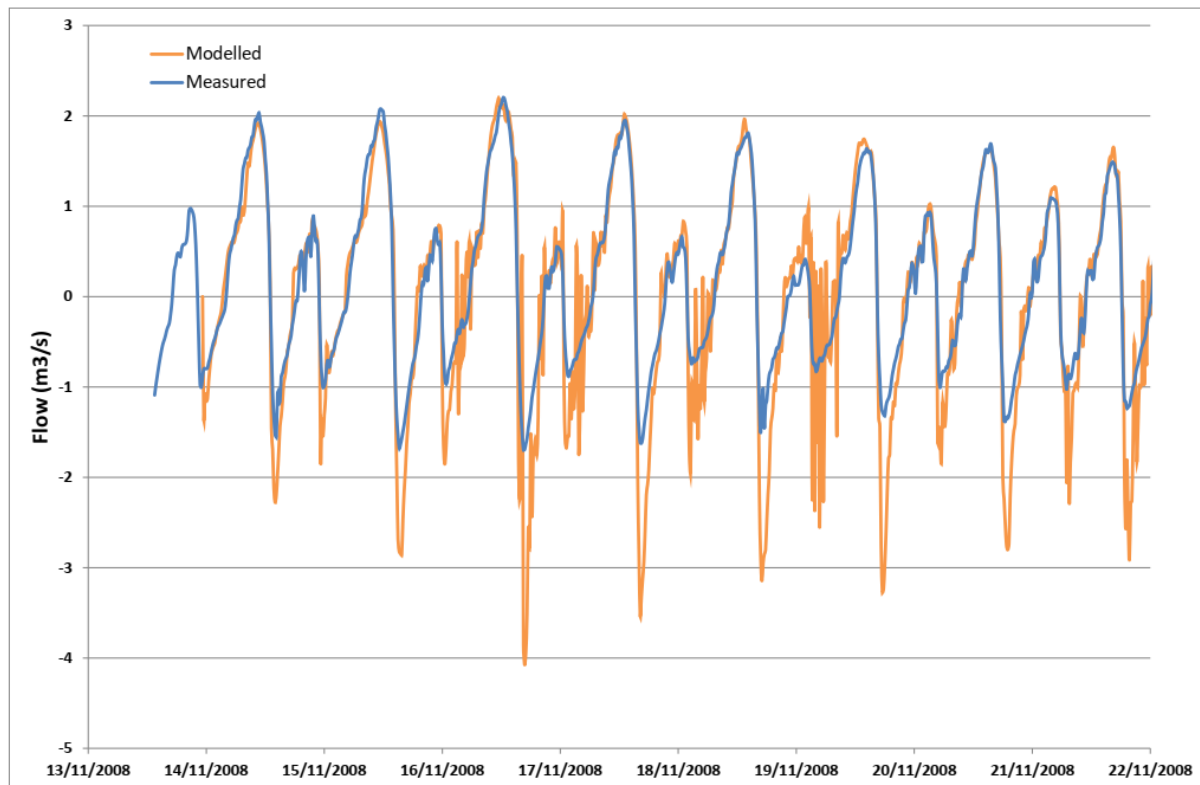

Supplement: S3 Fig — (PDF) [file pone.0254701.s003.pdf]
